# Does benralizumab effectively treat chronic obstructive pulmonary disease? A protocol of systematic review and meta-analysis

**DOI:** 10.1097/MD.0000000000020241

**Published:** 2020-05-15

**Authors:** Ru Chen, Ke-xin Wang, Xue Meng, Wen Zhou

**Affiliations:** a Department of Respiratory Medicine; b Department of Pediatrics, The Affiliated Hongqi Hospital of Mudanjiang Medical University; c Second Ward of Neurology Department, The Second Affiliated Hospital of Mudanjiang Medical University, Mudanjiang 157000, China.

**Keywords:** benralizumab, chronic obstructive pulmonary disease, efficacy, safety

## Abstract

**Background:**

This study aims to investigate the efficacy and safety of benralizumab for the treatment of patients with chronic obstructive pulmonary disease (COPD).

**Methods:**

This study will systematically and comprehensively search relevant literatures in electronic databases (MEDLINE, EMBASE, Cochrane Library, Global health, PsycINFO, Scopus, WANGFANG, and CNKI) from inception to the present without language and publication time restrictions. Two reviewers will independently carry out literature identification, data collection, and study quality assessment. Any disagreement will be settled down by a third reviewer through discussion and a consensus will be reached. RevMan 5.3 software will be used for statistical analysis performance.

**Results:**

This study will summarize up-to-date evidence to assess the efficacy and safety of benralizumab for the treatment of COPD.

**Conclusion:**

The findings of this study will provide helpful evidence to determine whether benralizumab is effective or not for the treatment of COPD.

**Systematic review registration:**

INPLASY202040039.

## 1 Introduction

Chronic obstructive pulmonary disease (COPD) is a preventable and treatable common lung disease.^[1,2,3,4]^ It often manifests as dyspnea, chronic cough, and sputum production.^[5,6]^ It is characterized by persistent respiratory symptoms and airflow limitation.^[7,8,9,10]^ It is reported that about 90% deaths related to COPD occur in Asia and Africa,^[11]^ and more than 0.9 million deaths are related to COPD.^[12]^ Thus, effective treatment for COPD is very important.

Benralizumab is a humanized, afucosylated monoclonal antibody, which is utilized for reduction of sputum and blood eosinophil counts.^[13,14,15,16,17]^ Previous studies have found that it can effectively treat patients with COPD.^[18,19,20]^ However, no systematic review has been conducted to examine the efficacy and safety of benralizumab for COPD. Thus, this systematic review will assess the efficacy and safety of benralizumab for the treatment of COPD.

## 2 Methods

### 2.1 Study registration

This study has been registered on INPLASY202040039, and it has been reported based on the Preferred Reporting Items for Systematic Reviews and Meta-Analysis (PRISMA) Protocol statement guidelines.^[21,22]^

### 2.2 Eligibility criteria

#### 2.2.1 Types of studies

Only randomized controlled trials (RCTs) of benralizumab for the treatment of COPD will be included. However, we will exclude any other studies, such as animal studies, case report, case series, review, comments, non-clinical trials, uncontrolled trials, and quasi-RCTs.

#### 2.2.2 Types of participants

Any patient who was diagnosed as COPD will be included irrespective of sex, age, and severity of COPD.

#### 2.2.3 Types of interventions

In the experimental group, all patients who received benralizumab treatment will be included.

In the control group, all patients received any management without restrictions. However, if we identified any study that involved any forms of benralizumab as their comparator, we will exclude it.

#### 2.2.4 Type of outcome measurements

Primary outcome is lung function, which was measured by forced vital capacity or forced expiratory volume in 1 second or other relevant tools.

Secondary outcomes are proportion of participants who had COPD exacerbation, rescue medication use, 6-minute walk test, dyspnea levels, quality of life (as measured by Saint George Respiratory Questionnaire or other tools), and adverse events.

### 2.3 Search methods for the identification of studies

#### 2.3.1 Electronic database records searches

Electronic searches will be performed systematically and comprehensively for relevant studies in MEDLINE, EMBASE, Cochrane Library, Global health, PsycINFO, Scopus, WANGFANG, and CNKI. All these databases will be conducted from inception to the present regardless of their language and publication time. A search strategy sample of Cochrane Library is created (Table 1). Similar search strategies will be adapted and applied to other electronic databases.

**Table 1:**
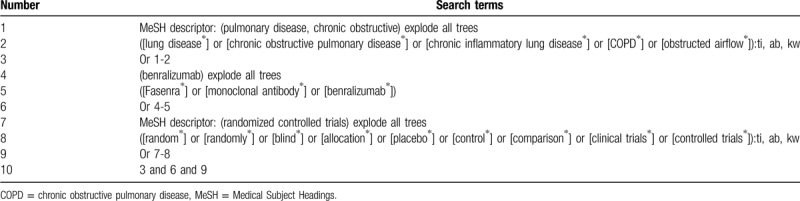
Search strategy for Cochrane Library.

#### 2.3.2 Searching other records source

To avoid missing potential studies, other record sources will be identified, such as conference abstracts, dissertations, and reference lists of included studies.

### 2.4 Data collection and analysis

#### 2.4.1 Study selection

Two reviewers will independently carry out study selection according to the previously designed eligibility criteria. Any disagreement will be solved by a third reviewer through discussion. Titles/abstracts of searched literatures will be identified to remove any irrelevant studies and duplicates. Then, we will read full text of remaining trials to further determine whether they meet all inclusion criteria. The whole process of study selection will be presented in a PRISMA flowchart.

#### 2.4.2 Data collection

Two reviewers will independently extract data based on the predefined data extraction sheet. A third reviewer will help to solve any discrepancies through discussion. We will collect data of title, first author, year of publication, region, race, gender, diagnostic criteria, eligibility criteria, trial setting, trial methods, details of interventions and controls, outcome indicators, results, findings, adverse events, follow-up information, and conflict of interest.

#### 2.4.3 Methodological quality assessment

Two reviewers will independently appraise study quality of all included trials using Cochrane Risk of Bias Tool, which covers 7 items, and each one is rated as low, unclear, and high risk of bias. We will invite a third reviewer to solve any different opinions by discussion.

#### 2.4.4 Dealing with missing data

Any unclear or missing data will be obtained from primary authors if possible. If we cannot request such data, we will analyze available data by intention-to-treat analysis.

#### 2.4.5 Data synthesis

RevMan 5.3 software will be utilized for performing statistical analysis. All discontinuous outcome variations will be estimated using risk ratio and 95% confidence intervals (CIs), and all continuous outcome variations will be calculated using weighted mean difference or standardized mean difference and 95% CIs. Statistical heterogeneity among included trials will be checked using *I*^2^ test: *I*^2^ ≤ 50% means minor heterogeneity, while *I*^2^ > 50% suggests considerable heterogeneity. A fixed-effects model will be applied to pool the data if *I*^2^ ≤ 50%. On the other hand, a random-effects model will be used to synthesize the data if *I*^2^ > 50%. When necessary, we will conduct a meta-analysis based on the similarity in characteristics of study and patient, interventions and controls, and outcome indicators. If obvious heterogeneity is identified, we will undertake a subgroup analysis to investigate possible sources of heterogeneity. In addition, we will report study results using narrative summary descriptions.

#### 2.4.6 Reporting bias

We will examine reporting bias using Funnel plot and Egger regression test if more than 10 RCTs are included.^[23,24]^

#### 2.4.7 Subgroup analysis

A subgroup analysis will be investigated to explore the possible sources of heterogeneity according to the different study characteristics, interventions, controls, and outcomes.

#### 2.4.8 Sensitivity analysis

A sensitivity analysis will be conducted to check robustness and stability of study findings by eliminating low-quality studies.

### 2.5 Dissemination and ethics

We expect to publish this study in a peer-reviewed journal. Ethical approval is not needed because no privacy patient data will be obtained.

## 3 Discussion

Although published studies have reported that benralizumab has been used for the treatment of COPD,^[18,19,20]^ there are still inconsistent results. In addition, no systematic review has been conducted to assess the efficacy and safety of benralizumab for the treatment of COPD. Therefore, this study will systematically investigate the efficacy and safety of benralizumab for the treatment of COPD. The findings of this study may provide evidence for clinical practice and health-related policy maker to improve COPD treatment approach.

## Author contributions

**Conceptualization:** Ru Chen, Xue Meng, Wen Zhou.

**Data curation:** Ru Chen, Ke-xin Wang, Xue Meng, Wen Zhou.

**Formal analysis:** Ke-xin Wang, Xue Meng.

**Funding acquisition:** Xue Meng, Wen Zhou.

**Investigation:** Wen Zhou.

**Methodology:** Ru Chen, Ke-xin Wang, Xue Meng.

**Project administration:** Wen Zhou.

**Resources:** Ru Chen, Ke-xin Wang, Xue Meng.

**Software:** Ru Chen, Ke-xin Wang, Xue Meng.

**Supervision:** Wen Zhou.

**Validation:** Xue Meng, Wen Zhou.

**Visualization:** Ru Chen, Ke-xin Wang, Xue Meng, Wen Zhou.

**Writing – original draft:** Xue Meng, Wen Zhou.

**Writing – review & editing:** Ru Chen, Ke-xin Wang, Xue Meng, Wen Zhou.
